# Supplementary material for: Mechanisms governing the pioneering and redistribution capabilities of the non-classical pioneer PU.1
Source: Nat Commun. 2020 Jan 21;11:402. doi: 10.1038/s41467-019-13960-2 (PMC6972792; doi:10.1038/s41467-019-13960-2)
Supplement: Supplementary file 7 — Source data [file 41467_2019_13960_MOESM7_ESM.zip › Source_Data/FigureS3/FigureS3J_MotifScanOutput/homerResults.html]

/misc/data/analysis/project\_PU1/predictionHepG2/motifs/ATAC\_PU1mutvsPU1.peaks.DESEQ/final - Homer de novo Motif Results


# Homer *de novo* Motif Results (/misc/data/analysis/project\_PU1/predictionHepG2/motifs/ATAC\_PU1mutvsPU1.peaks.DESEQ/final)

Known Motif Enrichment Results  
Gene Ontology Enrichment Results  
If Homer is having trouble matching a motif to a known motif, try copy/pasting the matrix file into
STAMP  
More information on motif finding results: HOMER
| Description of Results
| Tips
  
Total target sequences = 13198  
Total background sequences = 36498  
\* - possible false positive  

|  |  |  |  |  |  |  |  |  |
| --- | --- | --- | --- | --- | --- | --- | --- | --- |
| Rank | Motif | P-value | log P-pvalue | % of Targets | % of Background | STD(Bg STD) | Best Match/Details | Motif File |
| 1 | T A C G A T C G C A T G T C G A C G A T A C T G C G T A T A C G G C A T T G A C G C T A A G C T | 1e-1242 | -2.862e+03 | 45.48% | 12.54% | 289.3bp (203.9bp) | JunB(bZIP)/DendriticCells-Junb-ChIP-Seq(GSE36099)/Homer(0.994) More Information \| Similar Motifs Found | motif file (matrix) |
| 2 | C G A T C T A G G C A T C A G T C A G T C T G A G A T C G C T A A G C T C G T A C T A G | 1e-636 | -1.465e+03 | 45.27% | 20.21% | 307.7bp (199.7bp) | FOXM1(Forkhead)/MCF7-FOXM1-ChIP-Seq(GSE72977)/Homer(0.959) More Information \| Similar Motifs Found | motif file (matrix) |
| 3 | C A T G C A G T A G C T A G T C G T C A T C G A C T G A A C T G A C G T A G T C G A T C G C T A | 1e-426 | -9.816e+02 | 33.13% | 14.62% | 353.2bp (202.0bp) | HNF4G/MA0484.1/Jaspar(0.964) More Information \| Similar Motifs Found | motif file (matrix) |
| 4 | C A T G A G C T G C A T C G T A T G C A C G A T T C A G C G A T C A G T A C G T C T G A G T C A | 1e-222 | -5.122e+02 | 11.43% | 3.49% | 308.6bp (193.0bp) | HNF1b(Homeobox)/PDAC-HNF1B-ChIP-Seq(GSE64557)/Homer(0.935) More Information \| Similar Motifs Found | motif file (matrix) |
| 5 | C G T A C T A G A G T C G T C A T G C A C T G A A G C T T C G A G A C T A C G T C A G T C T A G G A T C | 1e-210 | -4.842e+02 | 13.11% | 4.65% | 307.7bp (192.1bp) | Arid5a/MA0602.1/Jaspar(0.794) More Information \| Similar Motifs Found | motif file (matrix) |
| 6 | C G T A T A C G G C T A C G A T T A G C G C T A T C G A C G T A T A C G T A C G A T C G | 1e-158 | -3.649e+02 | 15.48% | 7.16% | 331.4bp (195.5bp) | TCF7L2/MA0523.1/Jaspar(0.941) More Information \| Similar Motifs Found | motif file (matrix) |
| 7 | G C A T C A T G C T A G T G C A G T A C G C A T A C T G G A C T | 1e-154 | -3.561e+02 | 43.81% | 30.87% | 386.9bp (205.0bp) | TEAD3/MA0808.1/Jaspar(0.839) More Information \| Similar Motifs Found | motif file (matrix) |
| 8 | A T C G C G T A T A C G G A T C G C A T G A C T C G T A A T C G G C A T A G T C G T C A | 1e-140 | -3.229e+02 | 36.42% | 24.74% | 387.6bp (207.2bp) | Bach2(bZIP)/OCILy7-Bach2-ChIP-Seq(GSE44420)/Homer(0.745) More Information \| Similar Motifs Found | motif file (matrix) |
| 9 | C A T G A C G T A G C T T A G C T G C A C T G A A C T G A T C G G A C T A G T C C T G A | 1e-133 | -3.078e+02 | 19.71% | 10.92% | 374.9bp (205.4bp) | Nr5a2(NR)/Pancreas-LRH1-ChIP-Seq(GSE34295)/Homer(0.955) More Information \| Similar Motifs Found | motif file (matrix) |
| 10 | A C G T A C T G G T C A T G A C G C A T T G A C C G T A T C G A A C T G T A G C G A T C G C A T | 1e-131 | -3.037e+02 | 23.29% | 13.82% | 401.9bp (202.8bp) | FOS::JUN/MA0099.2/Jaspar(0.715) More Information \| Similar Motifs Found | motif file (matrix) |
| 11 | A C G T A C G T C T A G G A T C C T A G G A T C G T C A C G T A A G C T | 1e-127 | -2.928e+02 | 14.37% | 7.07% | 327.8bp (198.8bp) | CEBPE/MA0837.1/Jaspar(0.960) More Information \| Similar Motifs Found | motif file (matrix) |
| 12 | C T A G G C A T C A G T C A G T C G T A G T A C C G T A A G T C C T G A T C G A C G T A T C A G T A G C | 1e-113 | -2.609e+02 | 34.29% | 23.94% | 341.8bp (202.4bp) | PB0168.1\_Sox14\_2/Jaspar(0.782) More Information \| Similar Motifs Found | motif file (matrix) |
| 13 | A C T G G T A C G C A T A G C T C G A T C A T G A C G T C G A T C G T A C G T A G A T C | 1e-111 | -2.573e+02 | 33.61% | 23.39% | 362.1bp (198.2bp) | PB0081.1\_Tcf1\_1/Jaspar(0.726) More Information \| Similar Motifs Found | motif file (matrix) |
| 14 | A C T G A C G T A G T C C T G A C G T A A G T C C G T A | 1e-104 | -2.406e+02 | 16.90% | 9.59% | 313.1bp (207.2bp) | Foxa2(Forkhead)/Liver-Foxa2-ChIP-Seq(GSE25694)/Homer(0.834) More Information \| Similar Motifs Found | motif file (matrix) |
| 15 | G T C A A T G C C T G A C T G A C G T A A G C T C T G A A C T G G A C T | 1e-90 | -2.094e+02 | 8.94% | 4.07% | 358.5bp (195.3bp) | MF0005.1\_Forkhead\_class/Jaspar(0.731) More Information \| Similar Motifs Found | motif file (matrix) |
| 16 | A G C T A C T G A G T C A C T G A C G T G T C A C G T A | 1e-72 | -1.676e+02 | 29.00% | 21.11% | 399.2bp (208.6bp) | CEBPD/MA0836.1/Jaspar(0.814) More Information \| Similar Motifs Found | motif file (matrix) |
| 17 | G C T A G T C A C G T A T C A G A G C T C G T A G T A C G C T A T A G C C G A T A T C G G C A T | 1e-70 | -1.619e+02 | 22.76% | 15.74% | 352.0bp (204.1bp) | PB0134.1\_Hnf4a\_2/Jaspar(0.693) More Information \| Similar Motifs Found | motif file (matrix) |
| 18 | G C T A C T A G A T C G G T C A G T C A C G A T C A T G G A T C | 1e-63 | -1.463e+02 | 28.63% | 21.27% | 376.7bp (196.4bp) | TEAD(TEA)/Fibroblast-PU.1-ChIP-Seq(Unpublished)/Homer(0.924) More Information \| Similar Motifs Found | motif file (matrix) |
| 19 | T A G C G C A T C G A T C G T A T C A G G C T A G A C T G T C A G T C A C T G A G T A C G C T A | 1e-61 | -1.411e+02 | 9.98% | 5.59% | 349.9bp (198.8bp) | Foxf1(Forkhead)/Lung-Foxf1-ChIP-Seq(GSE77951)/Homer(0.795) More Information \| Similar Motifs Found | motif file (matrix) |
| 20 | T G A C G C T A T A G C T C G A T A G C C G T A A T C G T A G C G A C T A G T C C G A T A C T G | 1e-51 | -1.184e+02 | 37.14% | 29.91% | 440.4bp (208.3bp) | POL009.1\_DCE\_S\_II/Jaspar(0.757) More Information \| Similar Motifs Found | motif file (matrix) |
| 21 | C T A G A G C T G A C T C G T A A G T C A C G T A G T C C G T A A G T C A C G T A C T G C G T A A G T C A G T C | 1e-46 | -1.064e+02 | 31.48% | 24.97% | 402.0bp (207.7bp) | Reverb(NR),DR2/RAW-Reverba.biotin-ChIP-Seq(GSE45914)/Homer(0.693) More Information \| Similar Motifs Found | motif file (matrix) |
| 22 | C G A T C A T G C T A G T C G A C T G A C G A T A C T G A G C T A G C T C A G T C A T G G A T C G A C T T G C A | 1e-45 | -1.045e+02 | 4.84% | 2.27% | 353.6bp (192.9bp) | TEAD3/MA0808.1/Jaspar(0.729) More Information \| Similar Motifs Found | motif file (matrix) |
| 23 | A G T C C G A T A C T G T C A G A T G C A G C T A C G T G C A T T C A G | 1e-45 | -1.044e+02 | 16.23% | 11.33% | 421.0bp (203.1bp) | Nr5a2/MA0505.1/Jaspar(0.723) More Information \| Similar Motifs Found | motif file (matrix) |
| 24 | T G A C C T A G A T C G A T G C G A T C A G T C A G T C C G A T A T G C A G T C G A T C G A T C G T A C | 1e-45 | -1.041e+02 | 15.19% | 10.45% | 553.5bp (191.5bp) | SP1/MA0079.3/Jaspar(0.922) More Information \| Similar Motifs Found | motif file (matrix) |
| 25 | A C G T C A T G G C T A A T C G T A C G A C G T G T A C C G T A G A T C G T A C | 1e-44 | -1.034e+02 | 19.16% | 13.90% | 388.7bp (204.4bp) | Atf7(bZIP)/3T3L1-Atf7-ChIP-Seq(GSE56872)/Homer(0.915) More Information \| Similar Motifs Found | motif file (matrix) |
| 26 | C A G T A C T G A C G T A C T G G A C T G A T C G T C A C A G T G A T C C A T G A C T G | 1e-44 | -1.028e+02 | 12.93% | 8.58% | 387.0bp (206.0bp) | MEIS1/MA0498.2/Jaspar(0.749) More Information \| Similar Motifs Found | motif file (matrix) |
| 27 | C A T G A G T C A C T G A T C G A C G T A G T C C G A T A T C G | 1e-43 | -1.010e+02 | 44.71% | 37.75% | 438.5bp (206.7bp) | Smad2(MAD)/ES-SMAD2-ChIP-Seq(GSE29422)/Homer(0.810) More Information \| Similar Motifs Found | motif file (matrix) |
| 28 | A G T C G C T A A T G C C G A T C T A G A G C T T G C A G T A C G C T A A C T G C G A T | 1e-42 | -9.892e+01 | 29.60% | 23.44% | 399.7bp (212.1bp) | PB0091.1\_Zbtb3\_1/Jaspar(0.630) More Information \| Similar Motifs Found | motif file (matrix) |
| 29 | A G T C A C G T A G T C A G T C C G A T A G T C A G T C A G T C | 1e-41 | -9.492e+01 | 19.38% | 14.29% | 473.0bp (193.3bp) | Sp5(Zf)/mES-Sp5.Flag-ChIP-Seq(GSE72989)/Homer(0.827) More Information \| Similar Motifs Found | motif file (matrix) |
| 30 | A C G T A G C T A C T G A G T C C T A G C G A T G T A C C G T A | 1e-40 | -9.327e+01 | 16.16% | 11.52% | 375.0bp (196.8bp) | ATF4/MA0833.1/Jaspar(0.881) More Information \| Similar Motifs Found | motif file (matrix) |
| 31 | A C G T C A T G C G T A G A C T C T A G G T C A C G T A C G T A A G C T G A T C C G A T G A T C A T G C | 1e-40 | -9.265e+01 | 0.84% | 0.07% | 253.9bp (199.8bp) | Chop(bZIP)/MEF-Chop-ChIP-Seq(GSE35681)/Homer(0.821) More Information \| Similar Motifs Found | motif file (matrix) |
| 32 | G T A C G A C T A T G C C G A T A C T G T C G A T G C A T G A C A G T C C T A G | 1e-40 | -9.221e+01 | 24.20% | 18.69% | 432.9bp (203.0bp) | MF0004.1\_Nuclear\_Receptor\_class/Jaspar(0.709) More Information \| Similar Motifs Found | motif file (matrix) |
| 33 | C A G T A C T G T C G A G A T C A C G T A G T C G C T A A G C T A G T C A G C T A G T C | 1e-39 | -9.169e+01 | 5.05% | 2.56% | 357.2bp (203.0bp) | BATF::JUN/MA0462.1/Jaspar(0.794) More Information \| Similar Motifs Found | motif file (matrix) |
| 34 | T A C G A T G C G T A C C A T G A T C G T A G C C G T A T A C G T A G C A G T C C T A G A T C G | 1e-37 | -8.740e+01 | 15.10% | 10.74% | 506.8bp (198.9bp) | Tlx?(NR)/NPC-H3K4me1-ChIP-Seq(GSE16256)/Homer(0.672) More Information \| Similar Motifs Found | motif file (matrix) |
| 35 | C G T A T C G A A C T G A C T G A C G T A C G T C G T A | 1e-37 | -8.740e+01 | 15.10% | 10.75% | 348.2bp (201.0bp) | Nur77(NR)/K562-NR4A1-ChIP-Seq(GSE31363)/Homer(0.758) More Information \| Similar Motifs Found | motif file (matrix) |
| 36 | C A T G A T C G A T G C A G T C C G T A A C T G T A C G A T G C A G T C C G A T A C T G T A C G T A G C | 1e-37 | -8.595e+01 | 9.51% | 6.08% | 590.7bp (200.9bp) | Zfx/MA0146.2/Jaspar(0.628) More Information \| Similar Motifs Found | motif file (matrix) |
| 37 | G T C A A T G C C G T A C T A G C T A G T A C G A G T C A G C T A C T G | 1e-34 | -8.024e+01 | 27.13% | 21.75% | 490.4bp (206.7bp) | ZFX(Zf)/mES-Zfx-ChIP-Seq(GSE11431)/Homer(0.703) More Information \| Similar Motifs Found | motif file (matrix) |
| 38 | T C A G A C G T A C G T G T C A G T C A A G T C G A C T T G C A | 1e-33 | -7.735e+01 | 22.32% | 17.43% | 338.1bp (200.4bp) | PB0081.1\_Tcf1\_1/Jaspar(0.798) More Information \| Similar Motifs Found | motif file (matrix) |
| 39 | A T G C T G A C G C A T T C A G T A C G G T A C C T A G C T A G C A T G A T C G T A G C G A T C | 1e-32 | -7.508e+01 | 9.11% | 5.96% | 571.2bp (194.7bp) | Sp1(Zf)/Promoter/Homer(0.689) More Information \| Similar Motifs Found | motif file (matrix) |
| 40 | A C G T G A C T C G T A T A C G C G A T G T A C T G C A A G T C G C A T G C A T C G T A T C A G | 1e-31 | -7.334e+01 | 0.74% | 0.08% | 231.9bp (173.5bp) | FOSL1/MA0477.1/Jaspar(0.666) More Information \| Similar Motifs Found | motif file (matrix) |
| 41 | A C T G T A G C G A T C T A G C C A T G A T C G C T A G T A C G T A G C G A T C | 1e-31 | -7.291e+01 | 9.88% | 6.64% | 526.7bp (197.8bp) | TFAP2A/MA0003.3/Jaspar(0.728) More Information \| Similar Motifs Found | motif file (matrix) |
| 42 | T A G C A G T C A T C G A T C G A G T C A G T C A G T C | 1e-29 | -6.820e+01 | 13.75% | 10.05% | 615.3bp (176.1bp) | SP2/MA0516.1/Jaspar(0.770) More Information \| Similar Motifs Found | motif file (matrix) |
| 43 | G T C A A C T G G T C A A C T G A C T G A G T C A G T C | 1e-29 | -6.806e+01 | 25.82% | 20.95% | 440.1bp (202.3bp) | ZFX(Zf)/mES-Zfx-ChIP-Seq(GSE11431)/Homer(0.736) More Information \| Similar Motifs Found | motif file (matrix) |
| 44 | A C T G A C G T A C G T A C G T C T G A A C G T C G T A A G T C | 1e-28 | -6.626e+01 | 8.43% | 5.57% | 353.4bp (201.0bp) | Foxf1(Forkhead)/Lung-Foxf1-ChIP-Seq(GSE77951)/Homer(0.788) More Information \| Similar Motifs Found | motif file (matrix) |
| 45 | A T C G C G A T C A T G C G A T A C G T C A T G G T A C G T C A G C T A G A T C G T C A | 1e-28 | -6.571e+01 | 4.15% | 2.22% | 383.4bp (196.4bp) | CEBP:AP1(bZIP)/ThioMac-CEBPb-ChIP-Seq(GSE21512)/Homer(0.852) More Information \| Similar Motifs Found | motif file (matrix) |
| 46 | A T C G A C G T G C A T G C A T C T A G A G T C C T A G G A C T G C T A | 1e-27 | -6.414e+01 | 2.68% | 1.20% | 300.0bp (205.4bp) | Gmeb1/MA0615.1/Jaspar(0.655) More Information \| Similar Motifs Found | motif file (matrix) |
| 47 | A T C G T A G C C G A T T A C G G T C A G T A C G T C A C T G A T A C G A T G C G A T C | 1e-27 | -6.404e+01 | 26.09% | 21.35% | 425.5bp (206.1bp) | Tgif1(Homeobox)/mES-Tgif1-ChIP-Seq(GSE55404)/Homer(0.704) More Information \| Similar Motifs Found | motif file (matrix) |
| 48 | G C A T T A G C G T C A T G C A C T G A C A T G T A C G G C A T G C A T C T G A T A C G G C A T G A T C G C T A | 1e-26 | -6.116e+01 | 1.43% | 0.45% | 280.0bp (203.2bp) | Nr2f6/MA0677.1/Jaspar(0.675) More Information \| Similar Motifs Found | motif file (matrix) |
| 49 | G A T C C G A T C T A G G A C T T C G A G T A C A C G T C T A G C G T A G C T A A G C T C T G A | 1e-26 | -6.049e+01 | 1.44% | 0.46% | 455.3bp (198.8bp) | PB0178.1\_Sox8\_2/Jaspar(0.611) More Information \| Similar Motifs Found | motif file (matrix) |
| 50 | A C T G A C G T C G T A G T A C C G T A A T C G C G A T | 1e-26 | -6.018e+01 | 36.36% | 31.25% | 360.7bp (209.7bp) | Foxo3(Forkhead)/U2OS-Foxo3-ChIP-Seq(E-MTAB-2701)/Homer(0.668) More Information \| Similar Motifs Found | motif file (matrix) |
| 51 | A T G C G T A C G T A C C A T G T G A C C A T G C T A G T A C G T A G C | 1e-24 | -5.714e+01 | 13.50% | 10.13% | 544.7bp (194.1bp) | TFAP2A(var.2)/MA0810.1/Jaspar(0.831) More Information \| Similar Motifs Found | motif file (matrix) |
| 52 | A G T C C G A T A T C G G A C T A T G C C G T A T A C G T G A C T A C G | 1e-24 | -5.618e+01 | 31.08% | 26.36% | 427.6bp (211.4bp) | Meis1(Homeobox)/MastCells-Meis1-ChIP-Seq(GSE48085)/Homer(0.836) More Information \| Similar Motifs Found | motif file (matrix) |
| 53 | A G T C A G C T A C T G C G T A A C G T C G T A C G T A A T G C | 1e-23 | -5.526e+01 | 4.89% | 2.93% | 371.2bp (208.0bp) | PB0022.1\_Gata5\_1/Jaspar(0.910) More Information \| Similar Motifs Found | motif file (matrix) |
| 54 | C A T G T A C G A G C T A G C T A C G T C T A G A G C T C T G A A C T G A G T C A G T C A G C T C T G A | 1e-20 | -4.820e+01 | 0.50% | 0.06% | 227.5bp (181.9bp) | ZNF264(Zf)/HEK293-ZNF264.GFP-ChIP-Seq(GSE58341)/Homer(0.567) More Information \| Similar Motifs Found | motif file (matrix) |
| 55 | C T A G A T C G A C T G A T G C C G T A A C T G A T G C | 1e-20 | -4.804e+01 | 24.13% | 20.15% | 502.2bp (207.0bp) | MafA(bZIP)/Islet-MafA-ChIP-Seq(GSE30298)/Homer(0.792) More Information \| Similar Motifs Found | motif file (matrix) |
| 56 | C A G T C T A G A G C T T C G A A C T G G T A C A T G C A C G T C T G A C T A G C A T G C G T A C T A G A G T C | 1e-18 | -4.322e+01 | 0.52% | 0.08% | 516.8bp (212.8bp) | Rfx6(HTH)/Min6b1-Rfx6.HA-ChIP-Seq(GSE62844)/Homer(0.609) More Information \| Similar Motifs Found | motif file (matrix) |
| 57 | A G C T C G T A C A T G C G T A G A C T C T A G C A T G A C G T T C G A A C G T C T G A T A C G A T G C | 1e-18 | -4.296e+01 | 0.46% | 0.06% | 195.1bp (172.8bp) | PB0125.1\_Gata3\_2/Jaspar(0.598) More Information \| Similar Motifs Found | motif file (matrix) |
| 58 | C T G A T G A C C G A T C A T G G C T A C G T A A G T C G T C A A G T C C G A T A T C G G A C T T C G A | 1e-18 | -4.217e+01 | 1.46% | 0.60% | 291.8bp (212.4bp) | PB0099.1\_Zfp691\_1/Jaspar(0.674) More Information \| Similar Motifs Found | motif file (matrix) |
| 59 | A G C T G A T C C G T A C T A G G A C T C T G A G A T C C G T A C T A G G A C T T C G A G C T A A G T C T G C A | 1e-18 | -4.200e+01 | 0.68% | 0.16% | 245.3bp (202.4bp) | PB0051.1\_Osr2\_1/Jaspar(0.646) More Information \| Similar Motifs Found | motif file (matrix) |
| 60 | A G T C G A C T T C G A A C T G A C T G A G C T C T A G A C G T C T A G A G C T C T G A C T A G G A C T C T G A | 1e-18 | -4.199e+01 | 0.48% | 0.07% | 223.5bp (222.4bp) | TBX20/MA0689.1/Jaspar(0.718) More Information \| Similar Motifs Found | motif file (matrix) |
| 61 | A C T G A T G C A G T C A G C T A C G T A C T G A T G C C G A T A C T G C G T A C T A G A T G C A C G T T A C G | 1e-16 | -3.885e+01 | 0.43% | 0.06% | 166.6bp (216.0bp) | Zic(Zf)/Cerebellum-ZIC1.2-ChIP-Seq(GSE60731)/Homer(0.646) More Information \| Similar Motifs Found | motif file (matrix) |
| 62 | T G C A A G C T T G C A C T A G A C T G A T G C A G C T C T G A | 1e-16 | -3.806e+01 | 18.02% | 14.89% | 365.1bp (203.7bp) | Smad3(MAD)/NPC-Smad3-ChIP-Seq(GSE36673)/Homer(0.734) More Information \| Similar Motifs Found | motif file (matrix) |
| 63 | G T C A G A C T C T G A A G T C C T A G A G T C C G A T C T A G C T A G C G T A G T A C C T G A C T G A | 1e-16 | -3.787e+01 | 0.43% | 0.06% | 179.4bp (183.5bp) | ZNF416(Zf)/HEK293-ZNF416.GFP-ChIP-Seq(GSE58341)/Homer(0.619) More Information \| Similar Motifs Found | motif file (matrix) |
| 64 | G C T A G T A C C G T A C A T G T A G C G T C A T C G A G T A C C G A T A C T G A T G C G A T C G C A T | 1e-15 | -3.631e+01 | 18.60% | 15.50% | 377.6bp (203.0bp) | Smad3(MAD)/NPC-Smad3-ChIP-Seq(GSE36673)/Homer(0.559) More Information \| Similar Motifs Found | motif file (matrix) |
| 65 | A C T G A G T C A G T C A G T C A C G T A C T G A G T C A G T C A G T C G T C A A G T C C G T A A C T G C G T A | 1e-14 | -3.377e+01 | 8.24% | 6.20% | 475.1bp (205.0bp) | Sp1(Zf)/Promoter/Homer(0.641) More Information \| Similar Motifs Found | motif file (matrix) |
| 66 | A C T G A G T C A G C T A G T C G A T C C T A G G T A C A G T C A G T C C G T A C A T G C G A T A C G T | 1e-13 | -3.021e+01 | 0.89% | 0.33% | 184.7bp (237.7bp) | Sp5(Zf)/mES-Sp5.Flag-ChIP-Seq(GSE72989)/Homer(0.785) More Information \| Similar Motifs Found | motif file (matrix) |
| 67 | A C G T C G T A A C G T A C G T A C G T C G T A A G T C A G T C C G T A | 1e-12 | -2.950e+01 | 1.19% | 0.53% | 266.8bp (187.5bp) | FOXD2/MA0847.1/Jaspar(0.750) More Information \| Similar Motifs Found | motif file (matrix) |
